# Mechanism of Focal Adhesion Kinase Mechanosensing

**DOI:** 10.1371/journal.pcbi.1004593

**Published:** 2015-11-06

**Authors:** Jing Zhou, Camilo Aponte-Santamaría, Sebastian Sturm, Jakob Tómas Bullerjahn, Agnieszka Bronowska, Frauke Gräter

**Affiliations:** 1 Heidelberg Institute for Theoretical Studies, Heidelberg, Germany; 2 Leipzig University, Institute for Theoretical Physics, Leipzig, Germany; 3 Interdisciplinary Center for Scientific Computing (IWR), Heidelberg University, Heidelberg, Germany; University of Virginia, UNITED STATES

## Abstract

Mechanosensing at focal adhesions regulates vital cellular processes. Here, we present results from molecular dynamics (MD) and mechano-biochemical network simulations that suggest a direct role of Focal Adhesion Kinase (FAK) as a mechano-sensor. Tensile forces, propagating from the membrane through the PIP_2_ binding site of the FERM domain and from the cytoskeleton-anchored FAT domain, activate FAK by unlocking its central phosphorylation site (Tyr576/577) from the autoinhibitory FERM domain. Varying loading rates, pulling directions, and membrane PIP_2_ concentrations corroborate the specific opening of the FERM-kinase domain interface, due to its remarkably lower mechanical stability compared to the individual alpha-helical domains and the PIP_2_-FERM link. Analyzing downstream signaling networks provides further evidence for an intrinsic mechano-signaling role of FAK in broadcasting force signals through Ras to the nucleus. This distinguishes FAK from hitherto identified focal adhesion mechano-responsive molecules, allowing a new interpretation of cell stretching experiments.

## Author Summary

Focal adhesions integrate external mechanical signals into biochemical circuits allowing cellular mechanosensing. Although the zoo of mechanosensing proteins at focal adhesions is steadily growing, force-induced enzymatic mechanisms, as those uncovered for autoinhibited kinases in muscle, remain to be identified for focal adhesion downstream signaling. Here, we provide evidence that focal adhesion kinase (FAK) can act as a direct mechano-enzyme at focal adhesions, using molecular dynamics simulations and kinetic modelling. We show that anchorage of FAK to the membrane via PIP-2 is critical for this mechanical activation. Our results suggest similar mechanisms to be at play for other membrane-bound autoinhibited kinases.

## Introduction

Focal adhesions (FAs) act as key cellular locations for mechanosensing by integrating mechanical and biochemical signals between the outside and inside of the cell, thereby regulating processes such as cell proliferation, motility, differentiation, and apoptosis [1–3]. They contain numerous adapter or anchor proteins, which establish the mechanical link of the cytoskeleton with the extracellular matrix [4]. Some of these proteins have been identified as mechano-responsive elements [5–7].

**Fig 1:**
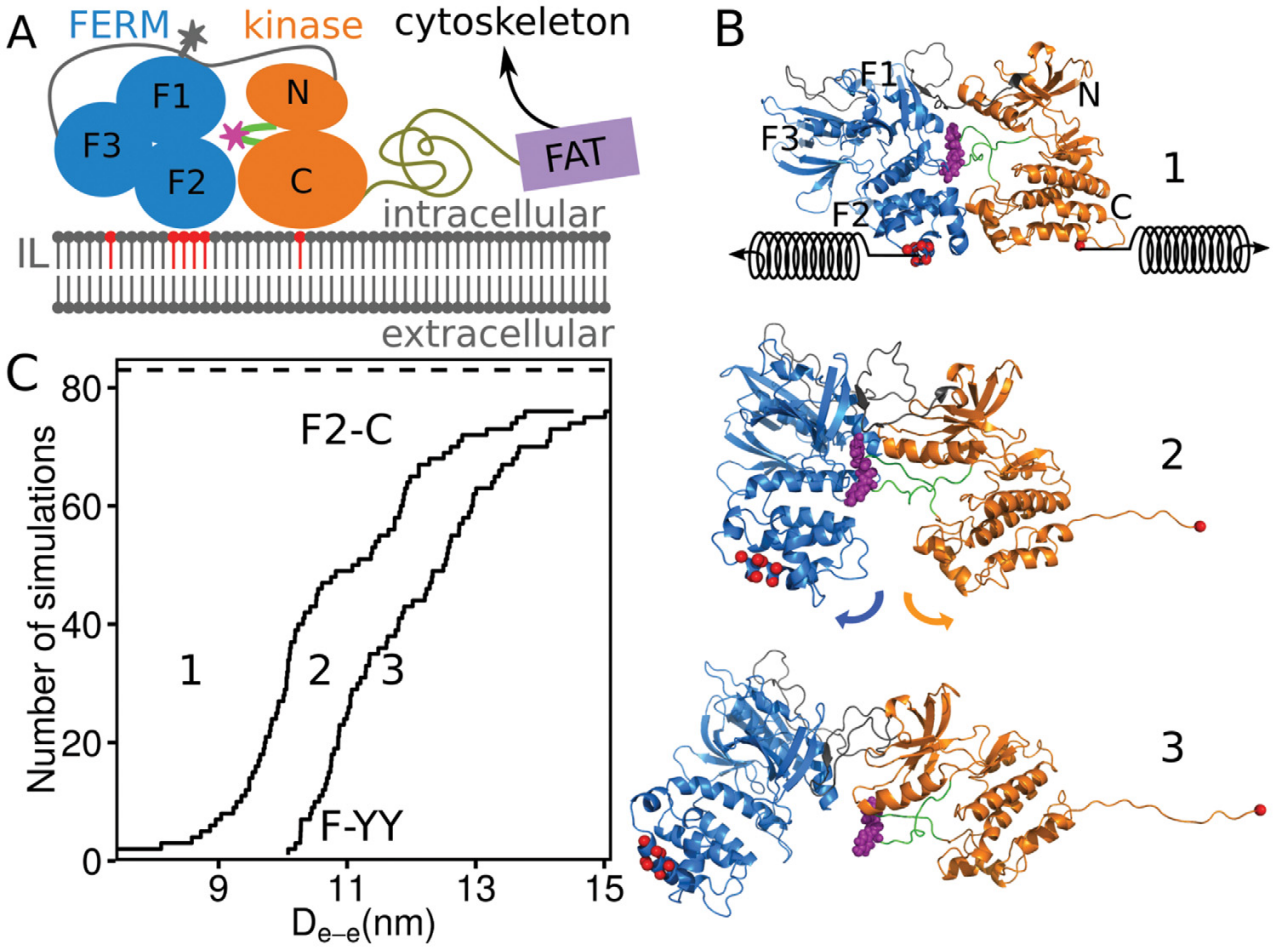
Mechanical activation of FK-FAK. A) Domain organization of FAK and its relative position at the cell periphery in the cytosol. The kinase domain (orange) contains the lobes N and C and the FERM domain (blue) consists of lobes F1–3, with F2 binding to PIP_2_ lipids (red) concentrated at the inner leaflet (IL) of the membrane (grey). The major phosphorylation site Tyr576/577 (magenta star), located at the activation loop (green) in the kinase domain, is autoinhibited by the FERM domain. The autophosphorylation site Tyr397 (grey star) is positioned in the loop connecting the kinase and FERM domains (grey). The FAT domain (violet) is not considered in our study. B) Stretching force applied to the basic patch in FERM and the kinase C-terminal residue (red), in form of virtual springs, induces FERM-kinase dissociation. Representative structures of FAK in its initial autoinhibited conformation (1), after dissociation of the kinase C-lobe from the FERM F2 lobe (2), and after Tyr576/577 release and partial C-terminal unfolding (3) are shown. Color-code and orientation of the protein as in A. C) Cumulative number of dissociation events as a function of the distance between the pulled elements at the moment of dissociation (D_*e*−*e*_). This indicates the extent of unfolding prior dissociation. Two events were monitored: dissociation of the the FERM F2 lobe from the kinase C-lobe, F2-C (transition from (1) to (2) in B), and separation of FERM domain from the Tyr576–577 phosphorylation site, F-YY (transition from (2) to (3) in B). Total number of simulations (83) is indicated with the dashed line.

Focal Adhesion Kinase (FAK) centrally regulates FAs by establishing adhesive interactions at the cell periphery [8]. Acting as a signaling hub between integrin and multiple proteins participating in downstream signaling pathways, it carries out diverse functions in embryonic development, cell migration, and survival, and its malfunction is associated with cancer progression and cardiovascular diseases [9, 10]. FAK comprises a central tyrosine kinase domain flanked by two large non-catalytic domains: FERM and FAT (Fig 1*A*). The N-terminal three-lobed 4.1 ezrin radixin moesin (FERM) homology domain is connected to the kinase N-lobe through a 50-residue linker. The C-terminal FAT (focal adhesion targeting) domain follows a 220-residue long proline-rich and disordered linker, through which it is connected to the kinase C-lobe. The activation of FAK first requires autophosphorylation of Tyr397, which offers a Src homology 2 (SH2) binding site. Src binding to FAK increases Src kinase activity, inducing the phosphorylation of Tyr576/577 within the kinase domain activation loop [11]. This is needed for maximal FAK-associated activity and leads to the formation of a Src-FAK complex, which triggers subsequent phosphorylations in the FAT domain and binding of downstream signaling proteins [12]. The FERM domain auto-inhibits the kinase domain by blocking the Tyr576/577 phosphorylation site [13, 14]. Exposure of this site is an essential step to permit its phosphorylation, and thereby render maximum FAK activity.

FAK locates at sites of integrin clustering through protein-protein interactions of its FAT domain, which contains binding sites for integrin- and actin-associated proteins [4, 15]. Integrin signaling and interactions with growth factor receptors were determined as FAK activators [16, 17]. Recent studies provided evidence that phosphoinositide phosphatidylinsositol-4,5-bis-phosphate (PIP_2_) is critical for efficient FAK activation and autophosphorylation [18, 19]. PIP_2_, a ubiquitous second messenger enriched in the inner leaflet of the plasma membrane and concentrated at FAs, regulates the interaction of cytoskeletal proteins with the membrane [20, 21]. PIP_2_ interacts directly with the basic patch (^216^KAKTLR^221^) in the FERM domain [18, 19], which induces conformational changes in FAK. The global concentration of PIP_2_ in the cell membrane is only approximately 1% [22]. However, PIP_2_-protein interactions [22, 23] or divalent ions, such as Ca^2+^, [24, 25] can lead to local PIP2 accumulation. Localized increments of Ca^2+^ were also suggested to increase the residency of FAK at FAs [26].

Evidence for a decisive role of FAK in mechanotransduction is steadily growing [27]. FAK is recruited to the leading edge and phosphorylated in migrating cells under shear stress [28, 29]. It has also been shown to mediate force-guided cell migration [30, 31] as well as strain-induced proliferation [32]. Recently, the mechano-sensitivity of FAK has been ascribed to the force-sensing fibronectin-integrin link [33]. However, until now, the available data on mechano-sensing through FAK is indirect. It remains unknown if FAK only lies downstream of mechano-sensing processes such as those involving integrins, or if FAK is also per se exposed to and activated by mechanical force.

We here hypothesize that mechanical force acts as a direct stimulus of FAK activity, indications for which are two-fold. First, FAK is tethered between the PIP_2_-enriched membrane and the cytoskeleton, likely acting as a force-carrying link in FAs. Second, the FERM-kinase structure suggests itself as a mechano-responsive scaffold, in which force could specifically detach the autoinhibitory FERM domain from the active site. FAK would be the first mechanoenzyme of FAs, allowing a direct transduction of a mechanical signal into an enzymatic reaction and downstream events into the nucleus, which would yield a mechanistic explanation of FAK’s mechano-sensing role [10]. Indeed, two analogous cases of mechanically activated enzymes have been previously identified, both of which are kinases and feature force-induced activation by removal of an autoinhibitory domain, namely titin and twitchin kinase in muscle [34–36]. In contrast to the FAT domain [37], the force response of the autoinhibited FERM-kinase fragment is currently unknown. To test the hypothesis of FAK as a force-sensor, we performed extensive equilibrium molecular dynamics (MD) and force-probe molecular dynamics (FPMD) simulations of the FERM-kinase fragment of FAK under various conditions. Force propagating onto FAK from a PIP_2_-enriched membrane and the cytoskeleton specifically opens the hydrophobic FERM-kinase interface, preparing FAK for activation via phosphorylation prior to the unfolding of the kinase domain. Given the low stability of the largely *α*-helical kinase and FERM domains, this is remarkable. The enforced activation is robust with regard to a large range of pulling velocities, but sensitive to the site of force application. Our force-induced activation pathway suggests a direct mechanoenzymatic function of FAK in FAs.

## Materials and Methods

Equilibrium MD simulations of the FERM-kinase fragment (FK-FAK) fragment [14] (PDB code: 2J0J) in the apo state, both in the absence and in the presence of a membrane containing PIP_2_ and POPE lipids, as well as of only the membrane, were performed using the GROMACS package [38]. FPMD simulations [39, 40] of FK-FAK without a membrane were performed by subjecting the C-terminal C-alpha atom and the center-of-mass of the C-alpha atoms of the basic patch ^216^KAKTLR^221^ to harmonic-spring potentials which were moved away from each other with constant velocity. FAT has been suggested to interact with FERM, binding to the same site as PIP2 does [41]. However, PIP2 binding is required for FAK activation [18, 19], thus excluding the possibility of FAT-FERM stable interactions for PIP2-mediated FAK activation. Other interactions between FAT and the FERM/Kinase complex are not known or at least suggested to be very dynamic and weak [42], and thereby easier to break under force conditions. This suggests that under tensile force, FAT is maintained sufficiently far from the complex, and that the force is transduced towards the complex through the fully-stretched 200 amino-acid proline-rich disordered linker. In consequence, in our FPMD simulations neither FAT nor the linker were considered.

The force response of membrane-bound FK-FAK was investigated by subjecting its C-terminus to a harmonic potential that was then moved away from the membrane either vertically or diagonally, while keeping the membrane position at its original position. PLS-FMA [43] was used to detect collective motions maximally correlated with the opening of the FERM-kinase interface. The underlying free energy landscape was characterized by analyzing the rupture forces as a function of loading rate, using both the HS model by Hummer & Szabo [44] and the BSK model by Bullerjahn et al. [45]. Kinetic models were based on previous biochemical networks [46–49] and simulated using COPASI [50]. Details of the methods are given in S1 Text.

## Results

### Force-induced release of FAK autoinhibition

*In vitro*, phosphorylation of the activation loop of FAK is enhanced by relieving the autoinhibition through Y180/M183 mutation [14] or PIP_2_-binding [18]. Catalytic turnover of wild-type FAK, however, requires an additional biochemical stimulus. Here, we ask if mechanical force could promote full domain dissociation of FAK as required for auto- and Src-phosphorylation–analogous to the effect of the Y180/M183 mutation. We examined the effect of tensile force on the autoinhibited FK-FAK using FPMD simulations. Tethering FAK between the membrane and the cytoskeleton results in force transmission from the membrane onto the basic patch of the FERM domain and from the paxillin-interacting FAT domain through the proline-rich linker onto the kinase C-terminus (Fig 1*A*). Accordingly, in our simulations, a pulling force was applied to the basic patch of FERM and the C-terminus of the kinase domain in opposite directions with 13 different pulling velocities from 6×10^−3^ nm/ns to 1 nm/ns (1 in Fig 1*B*). For each pulling velocity, multiple runs were carried out (83 runs in total), yielding a concatenated simulated time of about 7 *μ*s, with the slowest pulling simulation covering 1 *μ*s. We observed the autoinhibitory FERM domain to dissociate from the kinase domain in 76 out of 83 FPMD simulations (more than 90% of the cases). Conformational damage of either the FERM F2-lobe or kinase C-lobe occurred in the remaining 7 simulations. Release of Tyr576/577 from FERM occurred always later, i.e. at larger end-to-end distances, than dissociation of the F2-lobe from the C-lobe (Fig 1*B* and 1*C*), suggesting the F2-C detachment to be a requirement for mechanical FAK activation. We observed partial unfolding at the kinase C-terminus prior to exposure of Tyr576/577 to an only minor extent and mostly at higher loading rates, comprising at most a 15 nm increase in end-to-end length (Fig 1*B* and 1*C*), or no more than 30 residues of the C-terminal *α*-helix (S1 Fig). As the second half of this helix (or more) is typically disordered in other kinases (e.g. in protein kinase A or Src), its partial unfolding under force is likely not to impair FAK enzymatic function. Hence, our data suggest domain-domain dissociation to largely thwart the unfolding of the moderately stable *α*-helical domain structures. However, more substantial unfolding from the C-terminus of the kinase domain was the dominant pathway when pulling FK-FAK from its N and C-terminus (S2 Fig). Thus, we conclude that force acting specifically between the FERM basic patch and the kinase C-terminus removes the inhibitory FERM domain and thereby facilitates kinase activation, instead of domain unfolding and kinase inactivation.

### FAK-membrane interactions under force

**Fig 2:**
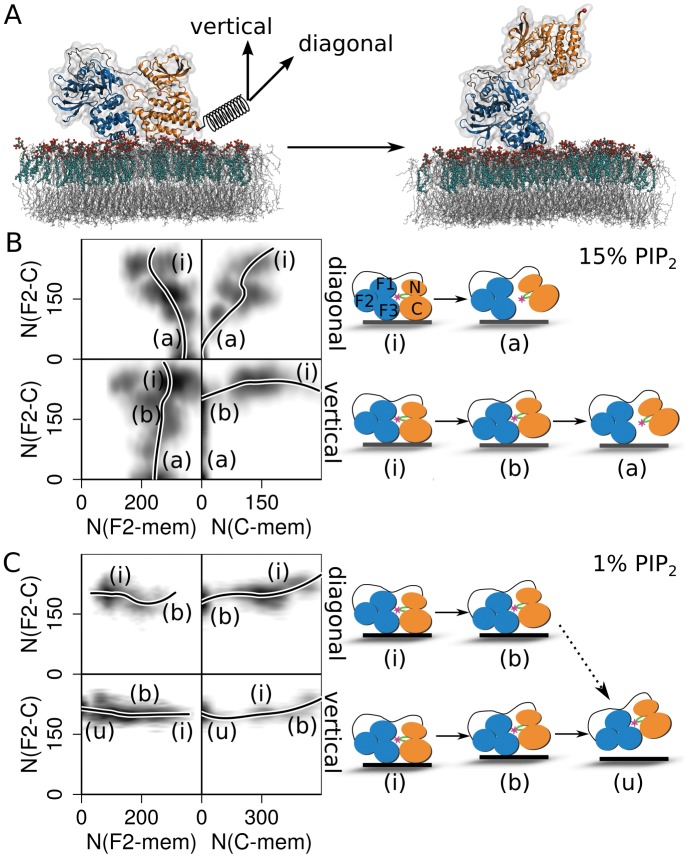
Mechanical activation of FAK bound to the membrane. A) Force was applied to the C-terminus of FK-FAK, in vertical or diagonal direction with respect to the membrane, with a counter-force acting on the membrane, leading to the release of autoinhibition (left to right transition). FK-FAK is shown as in Fig 1B, PIP_2_ lipids in the membrane (here at 15%) in cyan/red and POPE lipids in grey. B-C) Number of contacts *N* between the FERM F2-lobe (F2) and the kinase C-lobe (C) compared to the number of contacts between both lobes and the membrane (mem), at 15% (B) and 1% (C) PIP_2_ concentration. Number of contacts between lobes was defined as the number of atoms in one of the lobes closer than 0.6 nm to at least one atom of the other lobe. Upper panels show results for diagonal pulling while lower panels for vertical pulling. Densities of *N* (for a pulling velocity of 0.03 nm/ns) are shown as a grey gradient, with a polynomial fit to the data shown as a solid black line. The labels i, a, b, and u correspond to the inactive, active, bound and unbound states of FK-FAK, respectively, sketched at the right side.

At FAs, the specific interaction of the FERM basic patch with PIP2 is required for the anchoring of FK-FAK to the membrane. Other phospholipids only display background levels of binding [18]. In our previous study, we observed an allosteric change at the FERM-kinase interface upon PIP_2_ binding to FK-FAK, but no full opening [18]. This raised the question if full domain opening under force, as observed for isolated FK-FAK in solution, also occurs when FK-FAK is anchored to a membrane via PIP_2_. This would require both the PIP_2_-containing membrane as well as the PIP_2_-FERM link to be mechanically more robust against rupture than the FERM-kinase interaction. To test this, we set up a palmitoyloleoylphosphatidylethanolamine (POPE) membrane containing 15% (mol/mol) of PIP_2_ in the inner leaflet of the membrane, which was surrounded by water and neutralized by CaCl_2_. Within 100 ns of MD simulations starting from individual PIP_2_ molecules in the membrane, we observed the formation of small PIP_2_ clusters involving two or more lipids and Ca^2+^ (S3 Fig), accompanied by a decrease of area per lipid by ∼ 1 Å^2^ (S1 Table), in agreement with divalent-cation-mediated PIP_2_-enrichment in membranes [18, 24, 51]. FK-FAK was anchored to the membrane and the dynamics of the resulting complex was monitored over 150 ns of MD. Anchorage further increased clustering. The protein remained stably bound to the membrane through the FERM-PIP_2_ and additional interactions between the kinase C-lobe and the membrane, independent from the initial orientation of the protein relative to the membrane plane (Fig 2*A* *left* and S3 Fig). The same was observed for a membrane with 1% PIP_2_, which, however, showed less clustering and provided only a single PIP_2_ lipid for anchorage of FK-FAK.

Next, we monitored the mechanical response of membrane-anchored FK-FAK. In FPMD simulations, we subjected the protein to force by moving a harmonic spring attached to the kinase C-terminus with constant velocity along a direction vertical or diagonal to the membrane, while position restraining the center-of-mass of the membrane bilayer (Fig 2*A*). At 15% PIP_2_ concentration, independent of the pulling direction, we observed a loss of contacts of the kinase domain with the membrane and with the FERM domain, while the FERM-membrane interaction remained intact (Fig 2*B*). While diagonal pulling led to a concurrent dissociation of the kinase from the membrane and the FERM domain, vertical pulling resulted in kinase-membrane dissociation prior to kinase-FERM dissociation. In none of these simulations, we observed kinase unfolding prior to dissociation. Also, for both pulling directions, the membrane and the PIP_2_-FERM interaction were mechanically more robust than those at the FERM-kinase interface. Thus, the membrane simulations reproduced the process predominantly observed for isolated FK-FAK in solution (compare Fig 2*B* with Fig 1*B* and 1*C*). Namely, they all showed force-induced removal of the autoinhibitory FERM domain and exposure of the activation loop carrying the Tyr576/577 phosphorylation site.

When we applied force to FK-FAK anchored to a membrane containing only 1% PIP_2_, i.e. to a single PIP_2_ molecule, detachment of the kinase domain from the membrane was followed by the detachment of also the FERM domain (Fig 2C). Full loss of membrane anchoring naturally stops force transmission and impedes activation. Thus, an interaction of the FERM basic patch with multiple PIP_2_, which is likely in PIP_2_-enriched membranes, is required for mechanical FK-FAK activation. This is in line with the fact that PIP5K overexpression increases and PIP5K knockdown decreases the open FAK conformation [19]. The pulling direction, instead, appears to be less relevant.

### Mechanism of force-induced FK-FAK opening

We next analyzed in further detail the dynamics underlying the force-triggered FK-FAK domain-domain rupture. Applying partial least squares functional mode analysis (PLS-FMA) [43] to the simulations of isolated FK-FAK in solution, we obtained a collective opening motion that maximally correlates with the increase in minimal distance between the F2 and C lobes (S4 Fig). This opening motion also strongly correlated with the F2-C lobe distances obtained for the trajectories of membrane-bound FK-FAK, suggesting that it captures the essential opening dynamics of FK-FAK both isolated and bound to the membrane. This implies the simplified system of isolated FK-FAK in solution to follow a FERM-kinase dissociation mechanism, which is highly similar to the one of the more realistic system including the membrane, even though it lacks effects from FERM/kinase-membrane interactions.

**Fig 3:**
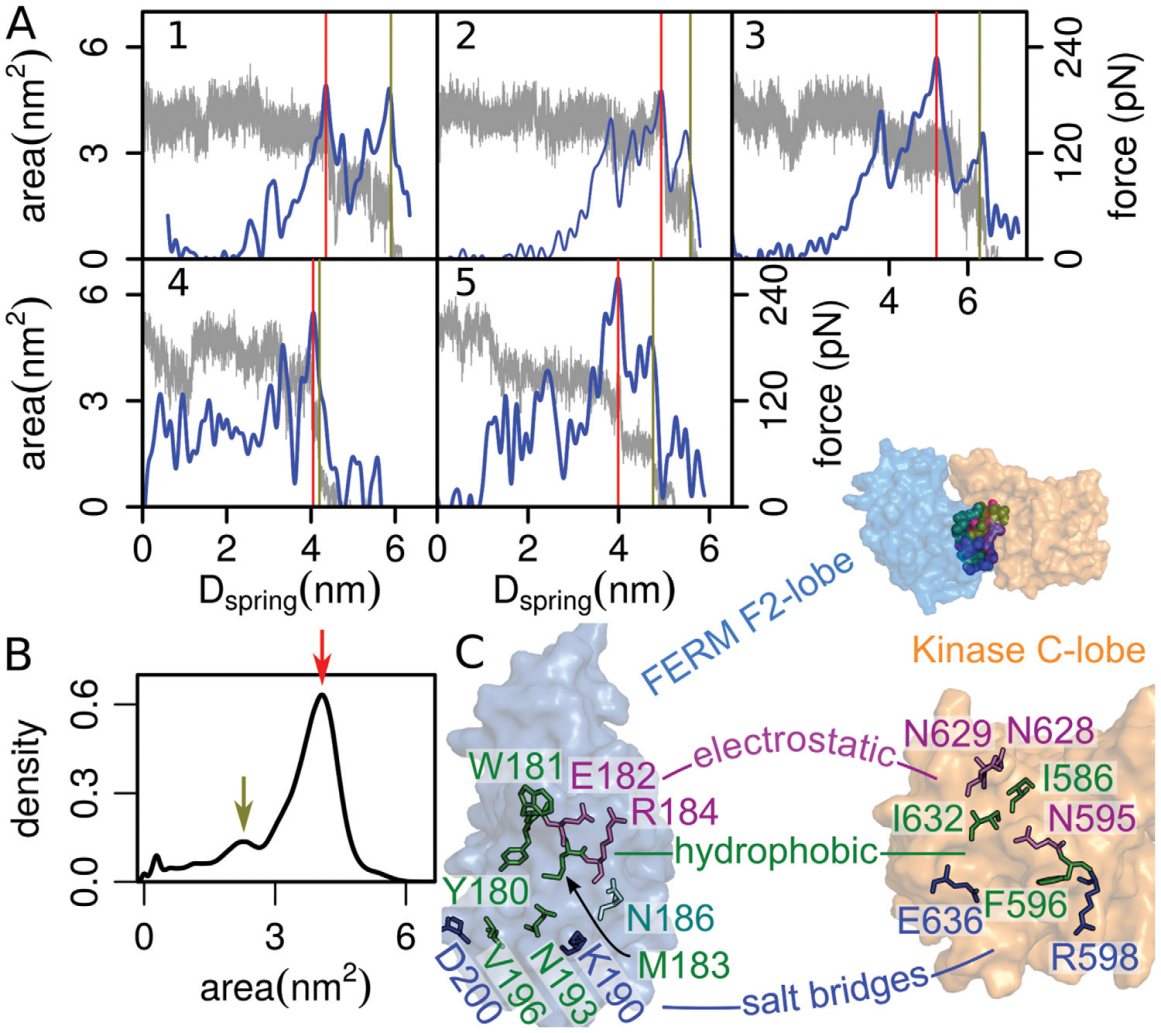
Mechanism of FK-FAK mechanical activation. A) Interfacial area between the F2- and C-lobe (grey) and average force exerted by the two springs (blue) as a function of the distance between springs, D_spring_. Results from six independent FPMD simulations are shown: (1–3) without the membrane pulling at *V* = 0.006, 0.006 and 0.014 nm/ns, respectively, and (4 and 5) pulling diagonally away from the membrane at *V* = 0.03 and 0.05 nm/ns, respectively. The interfacial area drops from initial values of 3–4.5 nm^2^ to intermediate values of 1.5–2.8 nm^2^. Afterwards it decreases to zero. Rupture force (highest force peak) always corresponded to the first drop in the interfacial area (red line). The peak force associated to the second drop in the area is highlighted with the green line. B) Distribution of interface areas reflecting the two states of FK-FAK during its force-induced opening (highlighted with arrows). All FPMD simulations were considered to compute the distribution. C) Residues involved in the rupture steps are highlighted as sticks. FERM F2- and Kinase C-lobe are shown in surface representation. Rupture steps are associated to the disruption of hydrophobic interactions (green); salt bridges (blue) and other electrostatic interactions (magenta), and interactions with other partners (cyan). Residues were identified by TRFDA (S5 Fig). They are listed in S2 Table.

We then identified the first steps along the opening motion of FK-FAK giving rise to rupture forces. Fig 3*A* shows typical force profiles and F2/C-lobe interaction areas as a function of the spring locations recovered from the FPMD simulations. For both FK-FAK in isolation and bound to the membrane, and independent of the loading rate, we observed that the interface area between the two lobes was reduced in two steps, both of which coincided with noticeable force peaks. The maximal force was reached when the first decrease in inter-lobe area occurred (from 3–4.5 nm^2^ to 1.5–2.8 nm^2^). This led to a short-lived intermediate, as reflected by a second peak in the distribution of the F2/C-lobe interface area (Fig 3*B*), before the two lobes fully dissociated. We note that the intermediate becomes less evident for faster pulling velocities.

To pinpoint the load-carrying residue-residue interactions across the interface, we calculated the punctual stress of each residue, using time resolved force distribution analysis (TRFDA) [52], and thereby detected the loss of inter-lobe interactions during pulling (see S5 Fig, S2 Table, and S1 Text). Inter-lobe interactions which ruptured reproducibly at one of the two dissociation steps are highlighted in Fig 3*C*. The first major rupture step required the breakup of a hydrophobic cluster composed of residues Y180, M183, N193, V196, and F596, and of an additional salt bridge (D200-R598). Rupture of these interactions gave rise to the maximal force, thus stressing their critical stabilizing role. Our results are in agreement with the observation that mutations Y180A, M183A, and F596D result in constitutively active FAK with an open FERM-kinase interface [14, 18]. Residue pairs rupturing at the second step included residues of mostly electrostatic nature (E182, R184, K190 and N595, N628, N629, E636) and are located further away from the membrane anchor. This second rupture step is immediately followed by the opening of the remaining FERM-kinase interface established between the F1 and the N-lobe, including the exposure of Tyr576/577. Thus, the rupture process resembles a zipper-like mechanism, during which the FERM and kinase interface is sequentially opened. Herein, the membrane-proximal hydrophobic patch around F596 represents the most robust mechanical clamp to be opened first. Our PLS-FMA calculations further support this sequential mode of opening (S4*C* Fig).

### Force required for FAK activation

**Fig 4:**
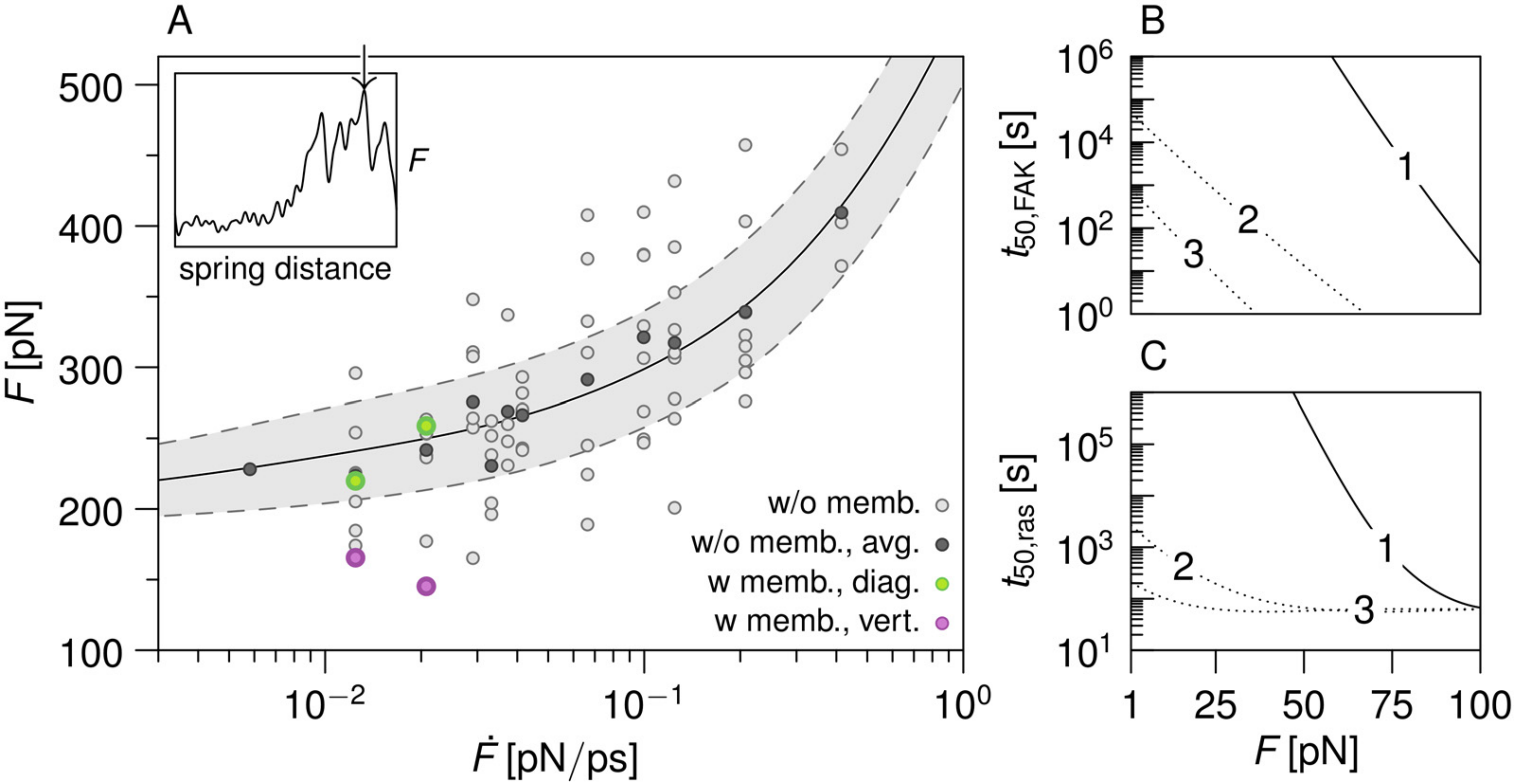
FAK mechano-signaling. A) Rupture force *F* as a function of the loading rate $\dot{F}$, where *F* is defined as the maximal force observed during FK-FAK activation (arrow in the inset). Light grey dots represent individual rupture forces *F* observed in our membrane-free FPMD simulations. Dark grey dots represent their averages (for each loading rate $\dot{F}$). The solid line shows the mean rupture force 〈*F*〉 predicted by the BSK model [45] for Δ*G* = 28.5 *k*_B_ *T*, *x*_b_ = 0.86 nm, and *D* = 6.6 × 10^6^ nm^2^/s. A fit with the HS [44] model yields similar model parameters (not shown). Dashed lines show the variation of the rupture forces predicted by the BSK model (2 standard deviations, see S1 Text for a detailed analysis). Pulling membrane-bound FK-FAK diagonally yielded similarly large rupture forces (green dots). Vertical pulling resulted in significantly lower rupture forces (pink dots), as this direction promotes the less resistant zipper-like dissociation mechanism described in S4B and S4C Fig) Time at which 50% of inactive FAK (B) and GDP-bound Ras protein (C) are consumed, under varying external force. Times obtained for three sets of parameters (1 to 3) corresponding to the three fits presented in S6 Fig.

Are the forces predicted by the simulations to relieve FAK autoinhibition relevant to FAK at FAs? At thirteen different loading rates, covering two orders of magnitude, we obtained maximal rupture forces for FK-FAK activation between 150 and 450 pN (Fig 4A). This force regime is similar to the one observed for titin kinase (400 pN at 0.2 pN/ps), a kinase known to be mechanically activated by forces present in muscle [35, 36, 53]. Our rupture forces are also similar to or slightly higher than those predicted by MD simulations of the focal adhesion proteins talin and vinculin (250–400 pN for nanosecond scale activation of talin [54, 55] and 100 pN for sub-nanosecond activation of vinculin [56], respectively). We then used both the HS model [44] and the BSK model [45] to fit the observed rupture forces as a function of the loading rate. This provided us with a set of compatible model parameters Δ*G*, *D* and *x*_b_, where Δ*G* denotes the activation energy, *D* the effective diffusivity and *x*_b_ the separation between the inactive state and the transition state. Focusing on those parameter combinations that correspond to a physiologically plausible spontaneous activation rate of no more than *k*_0_ = 10^−3^ Hz, we obtained a number of best-fit estimates from which we derived the force-dependent activation rate *k*(*F*) used in our kinetic model (Fig 4A and S6 Fig). We note that model parameters corresponding to an unphysiologically high spontaneous dissociation rate can improve our fit to the observed force fluctuations (S7 and S8 Figs). On this basis it might be speculated that there exists a second energy barrier at a larger value of *x*_b_ that guarantees thermal stability at low forces, but vanishes under the high forces used in our MD simulations. Nevertheless, this does not invalidate our qualitative findings on FK-FAK activation as force sensitivity increases exponentially with the barrier location *x*_b_ (see the S1 Text for a more detailed analysis).

## Discussion

We here provide computational evidence for a force-induced activation mechanism of FAK, in which tensile force relieves the blockage of its active site, as well as its central Tyr576/577 phosphorylation site, imposed by the autoinhibitory FERM domain. The release of autoinhibition is likely to make the kinase active site accessible for its substrate, Tyr397 of the same or another FAK molecule [42, 57], and/or to render Tyr576/577 accessible to Src. It is non-trivial that the exertion of a pulling force at opposite sites of the FERM and kinase domains leads to their dissociation. Other likely scenarios are protein unfolding and PIP_2_-protein dissociation, both of which would inactivate FAK, because an intact kinase structure and also PIP_2_ binding [18] are required for FAK activity. In fact, *α*-helical proteins are known to unfold at forces typically lower than *β*-sheet proteins [58], and both the kinase C-lobe and the FERM F2 domain feature mainly *α*-helical secondary structure. In this regard, force-induced FAK unfolding would be an expected result and was indeed preferred over FERM-kinase dissociation when pulling the FERM F1- or F3-lobe away from the kinase C-terminus. Instead, in the particular –and physiologically relevant– case that force is applied to the PIP_2_ binding site and the kinase C-terminus, we found domain-domain dissociation to be strongly preferred over unfolding or membrane detachment, over a large range of pulling velocities, and robust with regard to the pulling direction and presence of membrane interactions. Thus, the *α*-helical regions subjected to the pulling force mostly refrain from unfolding, and they instead transduce the load to the F2/C-lobe interface, which readily opens prior to substantial kinase unfolding. We suggest that it is the zipper-like topology, with the force application sites both located at the membrane-proximal basis of the two domains, that mechanically weakens the domain interface, resulting in efficient FAK opening and activation. Force transduction through the two termini, in contrast, results in shearing the two domains relative to each other, making them less prone to dissociate. The lower mechanical resistance of zipper versus shear-type topologies has been described earlier (e.g. [53]), and FAK force-induced domain-domain rupture and activation appears to be another variation of this theme.

Our findings not only decidedly argue for a mechano-sensing function of FAK, but also emphasize the crucial role of the membrane in mechanotransduction. First, membrane binding allows force to propagate to the F2-lobe of FAK. Second, the FAK-membrane interaction withstood the external load only in the case of PIP_2_-enriched membranes (15% PIP_2_). In contrast, FAK detached from low PIP_2_-content membranes. This supports the notion of PIP_2_ clustering as a requirement for FAK activation at FAs [18, 51]. We note that FAK can be activated *in vitro* by the sole action of PIP_2_ and Src, i.e. in the absence of tensile forces acting on membrane-bound FAK at FAs in stretched cells. However, it has become clear that FAK activation can proceed along different routes, depending on the cellular environment, and potentially can also involve pH changes [59], and/or growth factor receptors [16]. We here propose force to substitute or complement some of these activators shaping the multi-dimensional landscape of FAK activity.

Using TRFDA, we recovered the stabilizing role of Y180, M183, V196 and F596, a hydrophobic core previously shown by mutagenesis to stabilize the autoinhibited state [14], validating our simulation data. In addition, we found D200 and R598 to contribute to the rupture force, and predict their mutation to result in increased FAK activity.

The question arises, how force feeds into FAK-mediated Ras GDP/GTP exchange and regulates ERK-dependent gene expression, analogous to the chemical stimulation of growth factor receptor-dependent Ras signaling (S9 Fig) [27]. To assess how FAK as a mechanosensor couples mechanical signals into the downstream biochemical network, we defined force-dependent FAK activation as the initial step of a kinetic model for the Ras signaling pathway (S10 Fig) [46–49]. Both FAK opening and GDP/GTP exchange in Ras are accelerated by external forces, as expected (Fig 4*B* and 4*C* and S11*A* Fig). Intriguingly, while FAK force-induced activation shows a nearly linear dependency on force on the logarithmic scale (Fig 4*B*), Ras-GTP production shows a highly non-linear dependency and saturates beyond a critical force (Fig 4*C*). The reason is that activated FAK in complex with its partners, Grb2 and SOS via c-Src and SHC, acts as an enzyme for Ras activation. As a direct consequence, Ras activation follows mechano-enzymatic kinetics reminiscent of an inhibitory Michaelis-Menten mechanisms (S11*B* Fig) [60], in which force regulates the enzyme concentration.

In conclusion, our computational study provides direct evidence at the molecular level for a mechano-sensory role played by FAK at PIP_2_-enriched membranes of FAs. Through a specific domain opening mechanism regulated by force, FAK can integrate mechanical and chemical stimuli into downstream signaling to the nucleus. We suggest the mechano-enzymatics of FAK and Ras to provide a cap on the cell’s mechano-response. Our results, on FAK activation and signaling, are directly testable among others by molecular force sensors [61, 62] and cell stretching experiments. How other putatively mechano-activated kinases, such as the related Src kinase, follow similar mechanisms, at focal adhesions or elsewhere, remains to be shown.

## Supporting Information

S1 Fig Partial unfolding of the kinase domain. A) Distance between the pulled groups at the moment of dissociation (D_*e*−*e*_) as a function of the applied loading rate. Dissociation was monitored between the FERM F2-lobe and the kinase C-lobe (F2-C) and between the FERM domain and the Tyr576–577 phosphorylation site (F-YY). Each dot corresponds to one simulation run and the line is a polynomial fit to all the points as a guide to the eye, indicating a slight augment in D_*e*−*e*_ with increasing loading rate. B) Number of unstructured residues N_*UN*_ as a function of the time, for a simulation displaying a large D_*e*−*e*_ value (circle in A). Time trace is shown in black and a polynomial fit as a guide to the eye in blue. Residual unfolding of around 30 residues was observed at the C-terminus. The number of unstructured residues was obtained using VMD [63]. The inset shows the conformation of the Kinase domain at the moment of dissociation (at 42 ns, green line). Despite of the high loading rate, the conformation of the Kinase domain remained almost intact (unfolded residues of its C-terminus are highlighted in red).
(EPS)

S2 Fig Unfolding is preferred over dissociation of FK-FAK when exerting force to the FERM F1 or F3 lobes. The number of unfolded amino acids at the C-terminus of the FAK kinase domain is shown as a function of the number of contacts between the FERM F2- and the kinase C-lobe. Density recovered from the simulations is shown with the gray scale and polynomial fit with the black line. External force was applied to the N-terminus of the FERM F1-lobe (left) or the COM of the FERM F3-lobe (right). This is in contrast to the pathway observed in the majority of the simulations applying force to the basic patch of the FERM F2-lobe, in which only minor unfolding was observed before dissociation (up to 30 residues, see S1 Fig).
(EPS)

S3 Fig PIP_2_ interactions are mediated by calcium ions. Snapshots of a lipid bilayer in the absence (upper panels) and in the presence (lower panels) of FK-FAK. Snapshots were extracted from equilibrium MD simulations at the indicated times. 15% PIP_2_ lipids (yellow) are embedded in a palmitoyloleoylphosphatidylethanolamine (POPE) lipid bilayer (light red and periodic images in black). PIP_2_ formed clusters (dark red) mediated by Ca^2+^ ions (green). This caused a decrease in the area per lipid (see S1 Table). Clustering was less pronounced for lower PIP_2_ concentrations (see S1 Table). Lower right panel illustrates the FK-FAK bound to the bilayer, with the basic patch (cyan) at the FERM domain (blue) tethered to one of the PIP2-Ca^2+^ clusters (only this cluster is shown for clarity). The kinase domain (orange) also established interactions with the bilayer (red surface).
(EPS)

S4 Fig Partial least square functional mode analysis (PLS-FMA) to compare the force-induced dissociation mechanism of FK-FAK at different loading rates, in presence and absence of the membrane. A) Minimum distance between the F2- and C-lobe observed in FPMD simulations (black) compared to that predicted by PLS-FMA (color). To build the model (i.e. obtain the collective motion that is maximally correlated with the F2-C distance) the first half of the non-membrane FPMD simulations was considered (red). To validate the resulting model, the remaining part of these trajectories (green, model validation I), as well as, trajectories of FPMD simulations including membrane (blue, model validation II), were considered. B) Correlation coefficient between the minimum distance between the F2- and C-lobe predicted by PLS-FMA as a function of the number of PLS components. Same color coding as A. High correlation (> 0.9) was obtained both for the model and the the two validation data sets. Predictions in A correspond to the situation using 11 PLS components. C) Predicted ensemble-weighted motion of FK-FAK from its closed form (cyan) to its open form (gray), resembling a zipper-like motion.
(EPS)

S5 Fig Residue stresses during dissociation reveal critical residues for inter-domain stability of FK-FAK. Punctual stresses due to inter-lobe interactions of residues in the FERM domain (residues 175–245) and the kinase domain (residues 586–666) are shown as a function of spring distance during individual FPMD simulations. Punctual stresses were calculated using time resolved force distribution analysis (TRFDA, see S1 Text). Color coding according to the gray scale bar at the side. The red line indicates the distance between springs at which the highest force peak was observed. This event correspond to the transition of FK-FAK from the inactive to an intermediate state. The green line highlights the moment at which a second rupture event is observed, rendering FK-FAK activation. Punctual stresses are depicted for three FPMD simulations of FK-FAK isolated and in solution (A-C), at low pulling velocities, and for two FPMD simulations of FK-FAK bound to the membrane (D-E), pulling in diagonal direction from the membrane. Stresses are only shown for residues with significantly large stress values, and these residues varied among simulations. Residues that consistently showed pronounced drops in punctual stress upon rupture are listed in S2 Table and displayed in Fig 3C. of the main text.
(EPS)

S6 Fig Parameter space topography. A) least-squares fits of 〈*F*〉 to the data allow for a wide range of model parameters Δ*G* and *x*_b_. Fit computed via the BSK model. The HS model yields virtually identical results. Within the dark blue region, the RMSD error *δ*〈*F*〉 remains below 15 pN. As the effective transducer stiffness *κ*_*s*_ might be further reduced by the finite stiffness of the C-terminus and the FERM domain, we have successively reduced *κ*_*s*_ until we were no longer able to produce a good fit (lowest effective stiffness 0.01*κ*_*s*_ shown in orange). Black dotted lines show the force-induced increase in the unbinding rate at F = 50 pN, as evaluated using Eq. 6 of S1 Text. For the kinetic model, we only consider parameter combinations (*D*, Δ*G*, *x*_b_) that yield a reasonably low spontaneous dissociation rate *k*_0_ < 10^−3^ s^−1^ (evaluated for *D* = 10^6^ nm^2^/s. *k*_0_ = 10^−3^ s^−1^ boundary indicated by the red dashed line. B) representative rupture force fits, evaluated using the model parameters (and corresponding stiffnesses *κ*_*s*_ indicated in (A) and Fig 4 in the main text.
(EPS)

S7 Fig Unconstrained rupture force fitting. Dropping the requirement of thermal stability, we obtain a best-fit parameter set *D* = 1.2 × 10^7^ nm^2^/s, Δ*G* = 14.2*k*_*B*_ *T*, *x*_b_ = 0.3 nm that corresponds to unphysiologically large spontaneous activation rates *k*_0_ > 1 s^−1^, but produces a better fit to the observed rupture force spectra.
(EPS)

S8 Fig Bayesian parameter estimation. A) Lower panel: assuming a logarithmic prior distribution *p*_*i*_ = *p*_*i*,1_ ∝ [Δ*G* × *x*_b_ × *D*]^−1^ and integrating out *D*, we obtain a joint probability distribution for Δ*G* and *x*_b_ that is shown here for transducer stiffnesses of *κ*_*s*_ = 250 kJmol^−1^nm^−2^ (blue) and 0.1*κ*_*s*_ = 25 kJmol^−1^nm^−2^ (orange), respectively. Dark shaded areas enclose 50% of the corresponding parameter probability distributions, light shaded regions enclose 95%. Upper panel: marginal probability distributions for Δ*G* obtained by integrating out both *D* and *x*_b_. B) same as A, but evaluated for a uniform prior distribution *p*_*i*_(Δ*G*, *x*_b_, *D*) = *p*_*i*,2_(Δ*G*, *x*_b_, *D*) = const.
(EPS)

S9 Fig Kinetic scheme of FAK signaling regulated by external force. Numbering of individual reactions is arbitrary.
(EPS)

S10 Fig A scheme of the ERK/MAPK signaling pathway. Chemical signals such as EGF binding to RTK in the cell membrane induce autophosphorylation in the cytoplasmic domain of RTK. Then, through sequential phosphorylation of a number of downstream proteins, the signal is transduced into the cell nucleus. Mechanical signals act on the cell membrane and trigger the activation of the non-receptor tyrosine kinase, FAK, which is located at the cytoplasmic face of the membrane bilayer. Activated FAK thus mediates the signal transduction into the cell nucleus through a pathway constituted by a chain of similar interactions.
(EPS)

S11 Fig Downstream FAK signaling. A) Time course of FAK opening and the GDP/GTP exchange in the Ras protein obtained with varying external forces in a range between 1 and 100 pN (along the direction pointed by the blue arrow). Decrease in the concentration of closed FAK (black) and decrease in the concentration of GDP bound Ras protein (red) are shown. Time at which 50% of both GDP-bound Ras protein and closed FAK are consumed is highlighted with the dashed line. These values are shown as a function of applied force in Fig 4B and 4C of the main text. B) Rate of the GDP/GTP reaction as a function of the Ras-GDP concentration obtained for increasing external forces indicated with the blue arrow.
(EPS)

S1 Table Membrane structural properties at different PIP_2_ lipid concentrations. (EPS)

S2 Table Probability for residues in the FERM (left) and the kinase (right) domain to lose their interaction (in one of the two major dissociation steps, step 1 or 2, or elsewhere), based on TRFDA (S5 Fig). Residues dominating the first rupture step are labeled with * (hydrophobic interactions), ^‡^ (salt bridges) and ^§^ (varying partners), and residues dominantly involved in the second step in ^†^ (mostly electrostatic interactions). These residues are displayed in Fig 3*C* of main text.
(EPS)

S1 Text Supporting text. (PDF)
