# Supplementary material for: Mechanism of Focal Adhesion Kinase Mechanosensing
Source: PLoS Comput Biol. 2015 Nov 6;11(11):e1004593. doi: 10.1371/journal.pcbi.1004593 (PMC4636223; doi:10.1371/journal.pcbi.1004593)
Supplement: S1 Text — (PDF) [file pcbi.1004593.s014.pdf]

Supporting text S1 for:
Mechanism of Focal Adhesion Kinase Mechanosensing

Jing Zhou[1], Camilo Aponte-Santamaría[1], Sebastian Sturm[2], Jakob Tómas Bullerjahn[2], Agnieszka Bronowska[1], Frauke Gräter[1,3]

1 Heidelberg Institute for Theoretical Studies, Heidelberg, Germany
2 Leipzig University, Institute for Theoretical Physics, Leipzig, Germany
3 Interdisciplinary Center for Scientific Computing (IWR), Heidelberg University, Heidelberg, Germany

Molecular Docking

Partial atomic charges on the PIP_2 head group were assigned using the RESP methodology [1–3] and obtained from the REDS (RESP charge derive server [1, 2]), using the HF/6-31G* formalism (Hartree-Fock theory using a medium-sized basis set). The total charge on the PIP_2 head group was set to -5. The total charge of one PIP_2 lipid is -4 at pH 7. The remaining proton, however, is likely to be displaced by the positively charged Lys/Arg residues of the basic patch upon binding to FAK [4]. Molecular docking was carried out by using the UCSF DOCK6.5 suite [5], using the semi-rigid docking procedure and energy grid scoring in an implicit solvent. The grid spacing was 0.25 Å, and the grid included 1.2 nm beyond the FAK basic patch. The energy score was the sum of electrostatic and van der Waals contributions. In the course of the docking procedure, the PIP_2 molecule was subjected to 2500 cycles of molecular-mechanical energy minimization. The number of maximum ligand orientations was 5000. The best-scoring 25 FAK-PIP_2 complexes were further analyzed by means of molecular dynamics simulations. The best structure was chosen according to the favorable Coulombic interaction between the PIP_2 head group and the basic patch in the FERM domain.

Equilibrium molecular dynamics (MD) simulations

Three systems were simulated under equilibrium conditions: the FAK fragment consisting of the FERM and kinase domains as a single protein (FK-FAK), a membrane constituted by palmitoyloleoylphosphatidylethanolamine (POPE) and PIP_2 lipids, and the FK-FAK fragment in complex with such membrane. MD simulations were carried out using the GROMACS package 4.0.5 version [6], except from simulations including the membrane, which have been performed using GROMACS 4.5.5 [7].

The initial structure of the FK-FAK fragment (sequence region: 35-686) was taken from the crystal structure [8] (PDB code: 2J0J), completed by MODBASE [9] and equilibrated for 200 ns as described in our previous study [10]. The last snapshot of this simulation was considered as the initial conformation of the force-probe MD (FPMD) simulations of FK-FAK in the absence of the membrane.

To detect the effect of PIP_2 lipids on the force-induced FAK activation, different POPE lipid bilayers, with 1 %, 10 %, and 15 % PIP_2 concentrations in one of the leaflets, were simulated. The system was initially set up using the CHARMM graphical user interface [11]. 0.1 M NaCl was added to the system and the total charge of the system was neutralized by adding Ca^{2+} ions, which is required for PIP_2 clustering [12]. The lipid bilayer was solvated by explicit water molecules yielding a simulation system size of $15{\times}10{\times}11\,nm^3$. Bonds involving hydrogen atoms were constrained using the LINCS algorithm [13]. The temperature was kept constant at 300 K by using the velocity rescaling thermostat [14], with a coupling time of 0.1 ps. The pressure was kept constant at 1 bar by semi-isotropic coupling to a Parrinello-Rahman barostat [15] with a coupling time of 0.1 ps and a compressibility of $4.5\,bar^{-1}$. Electrostatic interactions were treated with the Particle Mesh Ewald [16] method using a grid spacing of 0.15 nm with a cubic interpolation. Short-range interactions were modeled through a Lennard-Jones potential. An integration

time step of 2 fs was used. The lipid bilayers (with different PIP_2 concentrations) were simulated for 100 ns of MD, by using the modified CHARMM36 force field [17] containing parameters of the PIP_2 lipid [18] and the TIPS3P water model [19]. In the presence of Ca^{2+}, PIP_2 lipids in the membrane are known to cluster and to form so-called lipid rafts [20]. To trace interactions between PIP_2 molecules, phosphor-phosphor (P-P) and phosphor-calcium (P-Ca) distances were calculated. If P-P or P-Ca was shorter than 0.6 nm and 0.4 nm, respectively, the two atoms were considered to interact. If a group of interacting atoms contained more than three phosphor atoms, it was defined as a microscopic cluster of aggregated PIP_2 [21]. The number of PIP_2 clusters and involved PIP_2 lipids per cluster were determined at each simulation step to monitor aggregation. We note that due to the limited system size, clustering here refers to microclusters of only few PIP_2 lipids, in contrast to the larger rafts observed commonly in experiments [22].

Equilibrium MD simulations of FK-FAK including the membrane were also carried out. The FERM domain was anchored via PIP_2 lipids to the membrane. ATP and Mg^{2+} were placed at the kinase domain in the orientation close to the one observed for the ANP in the crystal structure (PDB: 2J0L) [8]. In order to obtain an FK-FAK-PIP_2-membrane complex, the PIP_2 head group was docked to the FERM basic patch (^{216}KAKTLR^{221}) [10, 23] as described above. The head group of one of the PIP_2 lipids embedded in the bilayer was then replaced by the FAK PIP_2-head-group docked complex. The obtained complex was immersed in a triclinic water box ($15\times10\times19\,\mathrm{nm}^3$). 0.1 M NaCl was added to the system and the total charge of the system was neutralized by adding Ca^{2+} ions. The complex was equilibrated during 150 ns of MD simulations using the same simulation parameters and algorithms as described above for the membrane-only simulations. The last snapshot of this equilibration was considered as the starting conformation for the FPMD simulations of the FK-FAK in the presence of the membrane.

Force-probe MD (FPMD) simulations

FPMD simulations were performed on two different systems: on an isolated FK-FAK fragment in solution and on a FK-FAK fragment forming a complex with a membrane.

For the membrane-free FPMD simulations, the protein was accommodated at the center of a water box of size 18 nm × 10 nm × 10 nm. The protein OPLS all-atom force field [24] and the TIP4P water model [25] were used for these simulations. Simulation parameters and algorithms were the same as described above for the membrane-only simulations, except for an isotropic coupling to the barostat and a grid spacing of 0.12 nm for the electrostatic interactions. After 10000 energy minimization steps (using the steepest descent algorithm), the solvent was equilibrated during 2 ns of MD simulation with harmonic constraints on the protein heavy atoms with a force constant of k=1000 kJmol^{-1}nm^{-2}. We then attached the C-terminus of the kinase domain and the FERM basic patch to harmonic springs moving outwards at constant velocities V_1, V_2 (Fig. 1B of the main text). The applied force reads

$$F_i(t) = -\kappa_s \left[z_i(t) - V_i t \right], \tag{1}$$

where κ_s denotes the elastic constant of the pulling spring, $z_i(t)$ is the position of the center of mass of the kinase C-terminus (i=1) and the FERM basic patch (i=2) along the pulling coordinate z, and V_i is the velocity of the moving spring ($V_1 = -V/2$ and $V_2 = V/2$). V=0.006, 0.014, 0.03, 0.05, 0.07, 0.08, 0.09, 0.1, 0.16, 0.24, 0.3, 0.5 and 1 nm/ns were used. The measured forces F_i were averaged, $F = (|F_1| + |F_2|)/2$, and then low-pass filtered to decouple the pulling-induced changes in $z_i(t)$ from its fast intramolecular fluctuations. In the limit of low loading rates, the low-pass filtered pulling force can be evaluated analytically [26],

$$F \approx \frac{\kappa_s V t}{1 + \kappa_s/\kappa_m}, \tag{2}$$

where $\kappa_m = 2\Delta G/x_\mathrm{b}^2$ denotes the molecular stiffness. Although Eq. (2) becomes inaccurate as soon as the external pulling force approaches a critical force level $F_c = 2\Delta G/x_\mathrm{b}$ (see below), our fits indicate that F_c generally exceeds the observed rupture forces; we thus analyze all our data in terms of Eq. (2).

For the FPMD simulations of membrane-bound FK-FAK, the C-terminus of the kinase domain was pulled away from the membrane, either in a vertical (90°) or a diagonal (45°) direction to the membrane plane (Fig. 2*A* of the main text). The same harmonic forces were exerted on the pulled elements as in the FAK single protein simulation (Equation 1), but the kinase C-terminus was pulled at a speed of V=0.03 and 0.05 nm/ns, while the spring pulling the membrane was maintained at rest (V_2=0).

The number of contacts between two groups A and B was defined as the number of atoms in group A having a minimum distance of less than ((0.6 nm) to at least one of the atoms in group B (Fig. 2 in the main text). Dissociation events were assigned to the moment when the number of contacts dropped to zero. Dissociation of the FERM F2-lobe and the Kinase C-lobe was monitored (F2-C), as well as of the FERM domain and the Tyr576-577 phosphorylation site (F-YY). The separation between the pulled groups (D_{e-e}) was recorded at the moment of dissociation and is presented cumulatively for all FPMD runs in Fig. 1C of the main text.

The same force field was used for both the FPMD and equilibrium simulations of FK-FAK in the presence of the membrane, namely the modified CHARMM36 force field [17] including parameters for PIP2 lipid [18] and the TIPS3P water model [19]. Despite of the different force-field used for simulations with and without membrane, rupture forces in the presence of the membrane pulling diagonally were in the same range than those obtained in the absence of the membrane (Figs. 3A and 4A of main text). This indicates consistency between rupture-force data sets and their independence from the used force field. Reduction in the rupture forces when pulling vertically from the membrane (Fig. 3A of main text) thus appears to be feature attributed to a zipper-like dissociation pathway promoting less resistance and not to possible force-field inconsistencies.

Partial least squares functional mode analysis (PLS-FMA)

PLS-FMA [27] was carried out to identify similar dissociation motions of FK-FAK in the presence or the absence of the membrane. This method is a multiple linear least-square regression of the form $y(t) = X(t)b + e$. Here, a time-dependent functional variable y is expressed in terms of the time-dependent atomic positions X of a set of atoms, through a collective (PLS) vector b and residuals e. By minimizing the residuals e, the correlation between y and Xb is maximized. The PLS vector b is established as a linear combination of k uncorrelated regressors (PLS components), which are obtained via linear combinations of the original coordinates X with maximal covariance with y.

Here, the functional variable y was the separation between the FERM F2-and the kinase C-lobe, reflecting the closing-to-opening motion of FAK FERM-kinase fragment (black line in Fig. S4*A*). $X(t)$ were the time-dependent coordinates of backbone FAK FERM-kinase atoms. From the 43 FPMD trajectories in the absence of the membrane, we concatenated 21 of them to compute b, considering all possible loading rates (left in Fig. S4*A*). The remaining FPMD trajectories, in the absence of the membrane (middle in Fig. S4*A*) and also in the presence of the membrane (right in Fig. S4*A*), were used to validate the computed b. High correlation between y and X was obtained by using few PLS components (Fig. S4*B*), not only in the data set used to build the model, but also in the two independent data sets used to validate it. In the validation data sets, a correlation larger than 0.92 was obtained by considering 11 PLS components. The collective motion associated to the vector b in this situation is illustrated in Fig. S4*C*.

Time-resolved force distribution analysis

Time-resolved force distribution analysis (TRFDA) [28] was carried out to calculate the vector pairwise forces between residues as a function of simulation time. The pairwise force includes contributions from short-range Coulomb and Lennard-Jones potentials between residues ri in the FERM F2-lobe and rj in the kinase C-lobe and was calculated as:

$$\overrightarrow{F}_{ri,rj} = \sum_{i \in ri, j \in rj} \overrightarrow{F_{ij}} \tag{3}$$

where i is an atom of residue ri and j is an atom of residue rj. The sum of the absolute values of the vector pairwise forces acting on ri in the pulling direction z,

$$S_{ri} = \sum_{rj} |F_{z,ri,rj}|, \tag{4}$$

measures the stress acting on a residue through the domain interface along the direction of the external force. This stress is defined as punctual stress in units of force, and serves as a simple measure for where pairwise forces accumulate along the pulling direction. It was used here to identify residues involved either in the domain dissociation steps 1 or step 2. Note that the interactions between lobes were lost at the moment there were no more contacts between them and thereby the punctual stress decayed to zero. The TRFDA code is implemented in the Gromacs 4.5.3 package. Punctual stresses were calculated for the isolated FK-FAK, during FPMD simulations with pulling velocities V=0.006, 0.014, 0.03 nm/ns, and for FK-FAK-membrane complexes, during FPMD simulations with pulling diagonal to the membrane with pulling velocities V=0.03, 0.05 nm/ns (Fig. S5). TRFDA is a force-field based analysis. Forces here are directly derived from force-field interaction energies, which are dependent on the interatomic distance. Comparing with distances, forces are more sensitive to minor conformational distortion during partial domain-domain opening.. Another advantage of TRFDA is that it allows to monitor only the force projection along the pulling direction, which can directly reflect the force-induced changes at the domain-domain interface.

Rupture force fitting

The FK-FAK rupture forces $\langle F\rangle$ (obtained in the absence of a membrane) were fit as a function of the loading rate, using both the Hummer-Szabo (HS) [29] theory and a more comprehensive theory recently developed by Bullerjahn et al. (BSK) [30]. Both theories map the dissociating molecule to a fluctuating spring of stiffness κ_m and diffusivity D that breaks irreversibly once it reaches a maximum extension x_b relative to its rest length. In the absence of external force, the harmonic spring potential thus suppresses dissociation through a free energy barrier of height $\Delta G = \frac{1}{2}\kappa_m x_\mathrm{b}^2$, yielding a thermal dissociation rate that decreases exponentially in ΔG [29],

$$k_0 \sim \frac{2D\Delta G}{x_\mathrm{b}^2}\sqrt{\frac{\Delta G}{\pi k_\mathrm{B}T}}e^{-\Delta G/k_\mathrm{B}T}. \tag{5}$$

The free energy barrier ΔG can be reduced through the application of an external pulling force F, causing the force-dependent unbinding rate to increase exponentially in F [31],

$$\frac{k(F)}{k_0} = \left(1 - \frac{Fx_\mathrm{b}}{2\Delta G}\right)\exp\left\{\beta\Delta G\left[1 - \left(1 - \frac{Fx_\mathrm{b}}{2\Delta G}\right)^2\right]\right\}. \tag{6}$$

Eq. (6) is valid for constant forces F below the molecular breaking strength ($F \ll 2\Delta G/x_\mathrm{b}$). Refs. [29] and [30] generalize eq. (6) to higher forces and explicitly consider the force fluctuations exhibited by a pulling spring of finite stiffness κ_s.

Both the HS [29] and the BSK [30] model yield analytical predictions for the average rupture force $\langle F\rangle(\Delta G, x_\mathrm{b}, D, \kappa_s, v)$, where the physical parameters ΔG, x_b and D are a priori unknown whereas the pulling speed v and the stiffness κ_s of the pulling device are set by the experimentalist. In our simulations FK-FAK is extended symmetrically by two identical springs, each with a stiffness of $500\,\mathrm{kJ\,mol^{-1}\,nm^{-2}}$; this is equivalent to a single spring of half the stiffness, $\kappa_s = 250\,\mathrm{kJ\,mol^{-1}\,nm^{-2}}$. In principle, deformations of the C-terminus of the kinase domain might further reduce the effective transducer stiffness below this value; we therefore consider the value $\kappa_s^{\max} = 250\,\mathrm{kJ\,mol^{-1}\,nm^{-2}}$ only as an upper bound to the true linker stiffness. This slightly increases the uncertainty in our fit parameters, see Fig. S6.

To systematically assess the precision of our results, we varied the model parameters between $8k_\mathrm{B}T < \Delta G < 150k_\mathrm{B}T$, $0.05\,\mathrm{nm} < x_\mathrm{b} < 4\,\mathrm{nm}$ and $10^3\,\mathrm{nm}^2\,\mathrm{s}^{-1} < D < 10^8\,\mathrm{nm}^2\,\mathrm{s}^{-1}$ and tabulated the root-mean-square difference

$$\delta\langle F\rangle(\Delta G, x_\mathrm{b}, D) = \left[\frac{1}{N}\sum_{l=1}^{N}\left[\langle F\rangle(\kappa_s, v) - \langle F\rangle(\Delta G, x_\mathrm{b}, D, \kappa_s, v)\right]^2\right]^{1/2} \tag{7}$$

between the measured rupture forces $\langle F\rangle(\kappa_s, v)$ and the corresponding theoretical predictions $\langle F\rangle(\Delta G, x_\mathrm{b}, D, \kappa_s, v)$. We then projected $\delta\langle F\rangle(\Delta G, x_\mathrm{b}, D)$ onto the $(\Delta G, x_\mathrm{b})$-plane and plotted the region in parameter space that yielded an error $\delta\langle F\rangle$ no larger than 15 pN, see Fig. S6. Although this allows for large variations in the individual parameters ΔG and x_b, it turns out that our data restrict the possible parameter *combinations* $(\Delta G, x_\mathrm{b})$ to a narrow valley, see Fig. S6. Combining this with the physiologically motivated requirement of thermal stability (which we enforce by only considering parameters that yield a spontaneous dissociation rate k_0 no larger than $10^{-3}\,\mathrm{s}^{-1}$), we obtained a range of plausible parameter combinations $(D, \Delta G, x_\mathrm{b})$ that we used to predict the force-dependent increase $k(F)/k_0$ in the unbinding rate at physiological force levels, see Fig. S6. We furthermore used these parameter combinations and the force-dependent unbinding rate $k(F)$ derived therefrom as an input to our kinetic model for the Ras signalling pathway regulated by upstream FAK mechanical activation.

We also note that without the constraint on k_0, an alternative set of parameters (with $\Delta G \approx 14k_\mathrm{B}T$, $x_\mathrm{b} \approx 0.3\,\mathrm{nm}$) can be obtained that slightly improves the fit to our observed mean rupture forces $\langle F\rangle$, but greatly improves our fit to the observed rupture force fluctuations, see Fig. S7. To investigate this in detail, we analyzed our full rupture force distributions using the analytical rupture force distribution $p(\{F\} \mid \Delta G, x_\mathrm{b}, D, \kappa_s, v)$ provided by the BSK model. This distribution can be inverted to obtain from the experimentally observed rupture forces $\{F\}$ a probability distribution for the model parameters,

$$p(\Delta G, x_\mathrm{b}, D \mid \{F\}, \kappa_s, v) \propto p(\{F\} \mid \Delta G, x_\mathrm{b}, D, \kappa_s, v)\, p_i(\Delta G, x_\mathrm{b}, D). \tag{8}$$

The parameter distribution $p(\Delta G, x_\mathrm{b}, D \mid \{F\}, \kappa_s, v)$ depends on the (subjectively chosen) prior $p_i(\Delta G, x_\mathrm{b}, D)$; this dependence, however, is expected to vanish in the limit of large datasets when the conditional probability $p(\{F\} \mid \Delta G, x_\mathrm{b}, D, \kappa_s, v)$ becomes concentrated around a single, most probable point in parameter space [32]. We verify that a scale-free prior distribution $p_{i,1}(\Delta G, x_\mathrm{b}, D) \propto 1/[\Delta G \times x_\mathrm{b} \times D]$ and a uniform distribution $p_{i,2}(\Delta G, x_\mathrm{b}, D) \propto 1$ yield similar distributions for the underlying model parameters ΔG, x_b, D, and that these distributions concentrate around the previously found best-fit value at $\Delta G \approx 14k_\mathrm{B}T$, see Fig.S8.

On this basis, we might speculate that the observed rupture forces indeed derive from a relatively low energy barrier $\Delta G \approx 14k_\mathrm{B}T$ at a short distance $x_\mathrm{b} \approx 0.3\,\mathrm{nm}$, whereas the spontaneous dissociation behavior, and the rupture kinetics at physiological forces, might be controlled by a higher energy barrier $\Delta G^{>}$ at some distance $x_\mathrm{b}^{>} > x_\mathrm{b}$ large enough for the outer energy barrier to essentially vanish at the high pulling forces involved in our study, $x_\mathrm{b}^{>} - x_\mathrm{b} \gtrsim (\Delta G^{>} - \Delta G)/200\,\mathrm{pN}$. Although a quantitative measurement of the activation rate of FK-FAK at low forces would then be out of reach of MD simulations, we note that a hypothetical outer barrier would still be compatible with our findings; in particular, the requirement $(\Delta G^{>} - \Delta G)/200\,\mathrm{pN} < (x_\mathrm{b}^{>} - x_\mathrm{b})$ yields a similar set of allowed parameter values $(\Delta G^{>}, x_\mathrm{b}^{>})$ as shown in Fig. S6.

Kinetic modelling

Previous studies reported that Ras activation regulated by FAK, Src and Shc is essential for ERK activation [33, 34]. In the ERK/MAPK signaling cascade, signal transduction through FAK is achieved by multiple Grb2-mediated signaling pathways [34] (Figs. S9 and S10): (1) The SH2 domain of Shc binds to the autophosphorylated tyrosine site Tyr397 in FAK, and then is trans-phosphorylated by FAK. Phosphorylated Shc provides the binding site for Grb2. Subsequently, the FAK-Shc-Grb2 complex associates

with SOS and accelerates the exchange of GDP in Ras by GTP. (2) The SH2 domain of c-Src binds to the autophosphorylated tyrosine site Tyr397 in FAK and trans-phosphorylates Tyr925 in the FAT domain, which binds subsequently to Grb2. The FAK-Src-Grb2 complex then associates with SOS and contributes to the acceleration of the exchange of GDP/GTP in Ras protein. Our kinetic model of FAK-mediated downstream signaling is shown in Figs. S9 and S10. FAK-mediated signal transduction starts with the autophosphorylation of the tyrosine site of Tyr397 at the linker between the FAK FERM and kinase domain. Recent studies [23, 35] have shown that the clustering of FAK is critical for the autophosphorylation of Tyr397. Hence, the autophosphorylation reaction here was treated as a Michaelis-Menten mechanism with FAK as the enzyme. In our model, Tyr397 phosphorylation requires FAK opening by force. Thus, the newly introduced first step of the kinetic model is the conversion of closed inactive FAK into open active FAK by force, which was modeled by the DHS model using the MD-derived parameters (see above). Here, we focus on the effect of force-induced activation of FAK on the downstream signaling, thus, the activation mechanism of Src was simplified and only the step of autophosphorylation at Tyr416 was considered. The downstream steps of our proposed kinetic model are based on two existing mathematical models. The first model, put forward by Kholodenko et al, describes the signal transduction mediated by the EGF receptor [36], which comprises two Grb2-mediated signaling pathways identical to FAK signaling (Fig. S9). Except from the sequential phosphorylations in FAK (Tyr397, Try576/577 and Try925) after the force-induced domain opening, all parameters from Kholodenko's model were adopted to our model, including the thermodynamic restrictions along cyclic pathways in the reaction scheme (Fig. S9), e.g. $k_{10}\cdot k_{14}\cdot k_{16}\cdot k_{18}/k_{-10}\cdot k_{-14}\cdot k_{-16}\cdot k_{-18} = 1$. Secondly, parameters for the activation of the GDP/GTP exchange in Ras accelerated by the FAK-Grb2-SOS complex were taken from Hatakeyama's study [37], which describes this process with a Michaelis-Menten equation. All kinetic calculations were performed using COPASI [38]. Reactions, species and parameters are listed below.

List of Reactions for the kinetic modelling

#	Description	Rate equation	Ref.
1	[FAK] → [FAK$_o$]	$k_{01}\left(1-\frac{Fx}{2\Delta G}\right)\exp\left\{\beta\Delta G\left[1-\left(1-\frac{Fx}{2\Delta G}\right)^2\right]\right\}[FAK_o]$	[31]
2	[FAK$_o$] → [FAK$_o$-p]	k_{02cat}[FAK$_o$]^2/(k_{02m}+[FAK$_o$])	[39]
3	[Src] → [Src-p]	k_{03cat}[Src]^2/(k_{03m}+[Src])	[39]
4	[FAK$_o$-p] + [Src-p] ↔ [Src-P-FAK$_o$-p]	k_{04}[FAK$_o$-p][Src-p]-k_{04b}[Src-p-FAK$_o$-p]	[39]
5	[Src-p:FAK$_o$-p] → [Src-p-FAK$_o$-pp]	k_{05cat}[Src-p-FAK$_o$-p]	[39]
6	[Src-p:FAK$_o$-pp] → [Src-p:FAK$_o$-ppp]	k_{06cat}[Src-p:FAK$_o$-pp]	[39]
7	[FAK$_o$-p] + [Shc] ↔ [FAK$_o$-P-Sh]	k_{07}[FAK$_O$-p][Shc]-k_{07b}[FAK$_O$-p-Sh]	[36]
8	[FAK$_o$-p-Sh] ↔ [FAK$_o$-p:ShP]	k_{08}[FAK$_O$-p-Sh]-k_{08b}[FAK$_O$-p-ShP]	[36]
9	[FAK$_o$-p-ShP] ↔ [FAK$_o$-p] + [ShP]	k_{09}[FAK$_O$-p-ShP]-k_{09b}[FAK$_O$-p][ShP]	[36]
10	[Src-p-FAK$_O$-ppp] + [Grb2] ↔ [Src-p-FAK$_o$-ppp-G]	k_{10}[Src-p:FAK$_O$-p][Grb2]-k_{10b}[Src-p-FAK$_o$-p-G]	[36]
11	[ShP] + [Grb2] ↔ [ShP-G]	k_{11}[Shp][Grb2]-k_{11b}[ShP-G]	[36]
12	[ShP] → [Shc]	V_{12max}[ShP]/(k_{12m}+[ShP])	[36]
13	[FAK$_o$-p-ShP] + [Grb2] ↔ [FAK$_o$-p-ShP-G]	k_{13}[FAK$_o$-p-ShP][Grb2]-k_{13b}[FAK$_o$-p-Shc-G]	[36]
14	[Grb2] + [SOS] ↔ [G-S]	k_{14}[Grb2][Sos]-k_{14}[G-S]	[36]
15	[ShP-G] + [Sos] ↔ [ShP-G-S]	k_{15}[ShP-G][SOS]-k_{15b}[ShP-G-S]	[36]
16	[Src-p:FAK$_o$-ppp-G] + [SOS] ↔ [Src-p-FAKo-ppp-G-S]	k_{16}[Src-p-FAK$_o$-ppp-G][SOS]-k_{16b}[Src-p-FAKo-ppp-G-S]	[36]
17	[ShP] + [G-S] ↔ [ShP-G-S]	k_{17}[ShP][G-S]-k_{17b}[ShP-G-S]	[36]
18	[Src-p-FAK$_o$-ppp] + [G-S] ↔ [Src-p-FAKo-ppp-G-S]	k_{18}[Src-p-FAK$_o$-ppp][G-S]-k_{18b}[Src-p-FAK$_o$-ppp-G-S]	[36]
19	[FAK$_o$-p-ShP-G] + [SOS] ↔ [FAK$_o$-p-ShP-G-S]	k_{19}[FAK$_o$-p-ShP-G][SOS]-k_{19b}[FAK$_o$-p-ShP-G-S]	[36]
20	[FAK$_o$-p-ShP] + [G-S] ↔ [FAK$_o$-p-ShP-G-S]	k_{20}[FAK$_o$-p-ShP][G-S]-k_{20b}[FAK$_o$-p-ShP-G-S]	[36]
21	[FAK$_o$-p-ShP-G-S] ↔ [ShP-G-S] + [FAK$_o$-p]	k_{21}[FAK$_o$-p-ShP-G-S]-k_{-21}[ShP-G-S][FAK$_o$-p]	[36]
22	[Ras-GDP] → [Ras-GTP]; R-G-S	k_{22}[Ras-GDP](R-G-S)/(k_{22m} + [Ras-GDP])	[37, 40]
23	[Ras-GTP] → [Ras-GDP]	V_{23max}[Ras-GTP]/(k_{23m} + [Ras-GTP])	[37, 40]

List of Species for the kinetic modelling

Species	Name	Initial concentration	Ref.
FAK	Focal adhesion kinase	80 nM	[40, 41]
FAK_o	Opened FAK	0 nM	—
FAK_o-p	Opened FAK-py397	0 nM	—
Src	Src kinase	90 nM	[40, 41]
Src-p	Autophosphorylated Src-py416	0 nM	—
Shc	Shc adaptor protein	1000 nM	[37, 40]
Shc-P	phosphorylated Shc	0 nM	—
Grb2	Growth factor receptor bound protein 2	48 nM	[41]
ShP-G	Protein complex	0 nM	—
SOS	Son of Sevenless	62 nM	[41]
G-S	Grb2:SOS adaptor protein	0 nM	—
ShP-G-S	Protein complex	0 nM	
Src-p-FAK_o-p	Protein complex	0 nM	—
Src-p-FAK_o-pp	Protein complex (FAK-py397-py576/577)	0 nM	—
Src-p-FAK_o-ppp	Protein complex (FAK-py397-py576/577-py925)	0 nM	—
Src-p-FAK_o-ppp-G	Protein complex	0 nM	—
Src-p-FAK_o-ppp-G-S	Protein complex	0 nM	—
FAK_o-p-Sh	Protein complex	0 nM	—
FAK_o-p-ShP	Protein complex	0 nM	—
FAK_o-p-ShP-G	Protein complex	0 nM	—
FAK_o-p-ShP-G-S	Protein complex	0 nM	—
Ras-GDP	Ras protein (GDP state)	120 nM	[37, 40]
Ras-GTP	Ras protein (GTP state)	0 nM	—
R-G-S	Protein complex containing Grb2:SOS	0 nM	—

List of Parameters for the kinetic modelling

	Units	Model value	Ref. value	Ref.
k_{02cat}	s^{-1}	9.33	560 min^{-1}	[39]
k_{02m}	nM	0.132	0.132	[39]
k_{03cat}	s^{-1}	9.33	560 min^{-1}	[39]
k_{03m}	nM	0.132	0.132	[39]
k_{04}	$nM^{-1}s^{-1}$	0.09	0.09	[36]
k_{04b}	s^{-1}	0.6	0.6	[36]
k_{05}	s^{-1}	9.33	9.33	[39]
k_{06}	s^{-1}	9.33	9.33	[39]
k_{07}	$nM^{-1}s^{-1}$	0.09	0.09	[36]
k_{07b}	s^{-1}	0.6	0.6	[37]
k_{08}	$nM^{-1}s^{-1}$	6	6	[36]
k_{08b}	s^{-1}	0.06	0.06	[36]
k_{09}	s^{-1}	0.3	0.3	[36]
k_{09b}	$nM^{-1}s^{-1}$	0.0009	0.0009	[36]
k_{10}	$nM^{-1}s^{-1}$	0.003	0.003	[36]
k_{10b}	s^{-1}	0.05	0.05	[36]
k_{11}	$nM^{-1}s^{-1}$	0.003	0.003	[36]
k_{11b}	s^{-1}	0.1	0.1	[36]
k_{12m}	nM	340	340	[36]
V_{12max}	nMs^{-1}	1.7	1.7	[36]
k_{13}	$nM^{-1}s^{-1}$	0.003	0.003	[36]
k_{13b}	s^{-1}	0.1	0.1	[36]
k_{14}	$nM^{-1}s^{-1}$	0.0015	0.0015	[36]
k_{14b}	s^{-1}	0.0001	0.0001	[36]
k_{15}	$nM^{-1}s^{-1}$	0.03	0.03	[36]
k_{15b}	s^{-1}	0.064	0.064	[36]
k_{16}	$nM^{-1}s^{-1}$	0.01	0.01	[36]
k_{16b}	s^{-1}	0.06	0.06	[36]
k_{17}	$nM^{-1}s^{-1}$	0.021	0.021	[36]
k_{17}	s^{-1}	0.1	0.1	[36]
k_{18}	$nM^{-1}s^{-1}$	0.0045	0.0045	[36]
k_{18b}	s^{-1}	0.03	0.03	[36]
k_{19}	$nM^{-1}s^{-1}$	0.01	0.01	[36]
k_{19b}	s^{-1}	0.0214	0.0214	[36]
k_{20}	$nM^{-1}s^{-1}$	0.009	0.009	[36]
k_{20b}	s^{-1}	0.0429	0.0429	[36]
k_{21}	$nM^{-1}s^{-1}$	0.12	0.12	[36]
k_{21b}	s^{-1}	0.000214	0.000214	[36]
k_{22}	s^{-1}	0.222	0.222	[37]
k_{22m}	nM	0.181	0.181	[37]
k_{23m}	nM	0.0571	0.0571	[37]
V_{23max}	nMs^{-1}	0.289	0.289	[37]

References

1. Vanquelef E, Simon S, Marquant G, Garcia E, Klimerak G, Delepine JC, et al. R.E.D. Server: a web service for deriving RESP and ESP charges and building force field libraries for new molecules and molecular fragments. Nucleic Acids Res. 2011 Jul;39(Web Server issue):W511–W517. Available from: `http://dx.doi.org/10.1093/nar/gkr288`.

2. Dupradeau FY, Pigache A, Zaffran T, Savineau C, Lelong R, Grivel N, et al. The R.E.D. tools: advances in RESP and ESP charge derivation and force field library building. Phys Chem Chem Phys. 2010 Jul;12(28):7821–7839. Available from: `http://dx.doi.org/10.1039/c0cp00111b`.

3. Frisch MJ, Trucks GW, Schlegel HB, Scuseria GE, Robb MA, Cheeseman JR, et al.. Gaussian∼09 Revision D.01; 2009. Gaussian Inc. Wallingford CT.

4. McLaughlin S, Wang J, Gambhir A, Murray D. PIP(2) and proteins: interactions, organization, and information flow. Annu Rev Biophys Biomol Struct. 2002;31:151–175. Available from: `http://dx.doi.org/10.1146/annurev.biophys.31.082901.134259`.

5. Lang PT, Brozell SR, Mukherjee S, Pettersen EF, Meng EC, Thomas V, et al. DOCK 6: combining techniques to model RNA-small molecule complexes. RNA. 2009 Jun;15(6):1219–1230. Available from: `http://dx.doi.org/10.1261/rna.1563609`.

6. Hess B, Kutzner C, van der Spoel D, Lindahl E. GROMACS 4: Algorithms for highly efficient, load-balanced, and scalable molecular simulation. J Chem Theory Comput. 2008 Feb;4(3):435–447. Available from: `http://dx.doi.org/10.1021/ct700301q`.

7. Pronk S, Páll S, Schulz R, Larsson P, Bjelkmar P, Apostolov R, et al. GROMACS 4.5: a high-throughput and highly parallel open source molecular simulation toolkit. Bioinformatics. 2013 Apr;29(7):845–854. Available from: `http://dx.doi.org/10.1093/bioinformatics/btt055`.

8. Lietha D, Cai X, Ceccarelli DFJ, Li Y, Schaller MD, Eck MJ. Structural basis for the autoinhibition of focal adhesion kinase. Cell. 2007 Jun;129(6):1177–1187. Available from: `http://dx.doi.org/10.1016/j.cell.2007.05.041`.

9. Pieper U, Eswar N, Braberg H, Madhusudhan MS, Davis FP, Stuart AC, et al. MODBASE, a database of annotated comparative protein structure models, and associated resources. Nucleic Acids Res. 2004 Jan;32(Database issue):D217–D222. Available from: `http://dx.doi.org/10.1093/nar/gkh095`.

10. Zhou J, Bronowska A, Le Coq J, Lietha D, Gräter F. Allosteric regulation of focal adhesion kinase by PIP$_2$ and ATP. Biophys J. 2015 Feb;108(3):698–705. Available from: `http://dx.doi.org/10.1016/j.bpj.2014.11.3454`.

11. Wu EL, Cheng X, Jo S, Rui H, Song KC, Dávila-Contreras EM, et al. CHARMM-GUI Membrane Builder toward realistic biological membrane simulations. J Comput Chem. 2014 Oct;35(27):1997–2004. Available from: `http://dx.doi.org/10.1002/jcc.23702`.

12. Ellenbroek WG, Wang YH, Christian DA, Discher DE, Janmey PA, Liu AJ. Divalent cation-dependent formation of electrostatic PIP2 clusters in lipid monolayers. Biophys J. 2011 Nov;101(9):2178–2184. Available from: `http://dx.doi.org/10.1016/j.bpj.2011.09.039`.

13. Hess B, Bekker H, Berendsen HJC, Fraaije JGEM. LINCS: A linear constraint solver for molecular simulations. J Comput Chem. 1997;18(12):1463–1472. Available from: `http://dx.doi.org/10.1002/(SICI)1096-987X(199709)18:12<1463::AID-JCC4>3.0.CO;2-H`.

14. Bussi G, Donadio D, Parrinello M. Canonical sampling through velocity rescaling. J Chem Phys. 2007;126(1):014101 – 014101–7. Available from: http://link.aip.org/link/?JCP/126/014101/1.

15. Parrinello M, Rahman A. Polymorphic transitions in single crystals: A new molecular dynamics method. J Appl Phys. 1981;52(12):7182–7190. Available from: http://link.aip.org/link/?JAP/52/7182/1.

16. Darden T, York D, Pedersen L. Particle mesh Ewald: An Nlog(N) method for Ewald sums in large systems. J Chem Phys. 1993;98(12):10089–10092. Available from: http://link.aip.org/link/?JCP/98/10089/1.

17. Huang J, MacKerell AD Jr. CHARMM36 all-atom additive protein force field: validation based on comparison to NMR data. J Comput Chem. 2013 Sep;34(25):2135–2145. Available from: http://dx.doi.org/10.1002/jcc.23354.

18. Fuller JC, Martinez M, Wade RC. On calculation of the electrostatic potential of a phosphatidylinositol phosphate-containing phosphatidylcholine lipid membrane accounting for membrane dynamics. PLoS One. 2014;9(8):e104778. Available from: http://dx.doi.org/10.1371/journal.pone.0104778.

19. Jorgensen WL. Quantum and statistical mechanical studies of liquids. 10. Transferable intermolecular potential functions for water, alcohols, and ethers. Application to liquid water. Journal of the American Chemical Society. 1981;103(2):335–340. Available from: http://dx.doi.org/10.1021/ja00392a016.

20. Giannone G, Rondé P, Gaire M, Beaudouin J, Haiech J, Ellenberg J, et al. Calcium rises locally trigger focal adhesion disassembly and enhance residency of focal adhesion kinase at focal adhesions. J Biol Chem. 2004 Jul;279(27):28715–28723. Available from: http://dx.doi.org/10.1074/jbc.M404054200.

21. Cruz-Chu ER, Schulten K. Computational microscopy of the role of protonable surface residues in nanoprecipitation oscillations. ACS Nano. 2010 Aug;4(8):4463–4474. Available from: http://dx.doi.org/10.1021/nn100399f.

22. van den Bogaart G, Meyenberg K, Risselada HJ, Amin H, Willig KI, Hubrich BE, et al. Membrane protein sequestering by ionic protein-lipid interactions. Nature. 2011 Nov;479(7374):552–555. Available from: http://dx.doi.org/10.1038/nature10545.

23. Goñi GM, Epifano C, Boskovic J, Camacho-Artacho M, Zhou J, Bronowska A, et al. Phosphatidylinositol 4,5-bisphosphate triggers activation of focal adhesion kinase by inducing clustering and conformational changes. Proc Natl Acad Sci U S A. 2014 Aug;111(31):E3177–E3186. Available from: http://dx.doi.org/10.1073/pnas.1317022111.

24. Jorgensen WL, Tirado-Rives J. The OPLS [optimized potentials for liquid simulations] potential functions for proteins, energy minimizations for crystals of cyclic peptides and crambin. Journal of the American Chemical Society. 1988;110(6):1657–1666. Available from: http://dx.doi.org/10.1021/ja00214a001.

25. Jorgensen WL, Chandrasekhar J, Madura JD, Impey RW, Klein ML. Comparison of simple potential functions for simulating liquid water. J Chem Phys. 1983;79(2):926–935. Available from: http://link.aip.org/link/?JCP/79/926/1.

26. Maitra A, Arya G. Model accounting for the effects of pulling-device stiffness in the analyses of single-molecule force measurements. Phys Rev Lett. 2010 Mar;104(10):108301.

27. Krivobokova T, Briones R, Hub JS, Munk A, de Groot BL. Partial least-squares functional mode analysis: application to the membrane proteins AQP1, Aqy1, and CLC-ec1. Biophys J. 2012 Aug;103(4):786–796. Available from: http://dx.doi.org/10.1016/j.bpj.2012.07.022.

28. Costescu BI, Gräter F. Time-resolved force distribution analysis. BMC Biophys. 2013;6(1):5. Available from: http://dx.doi.org/10.1186/2046-1682-6-5.

29. Hummer G, Szabo A. Kinetics from nonequilibrium single-molecule pulling experiments. Biophys J. 2003 Jul;85(1):5–15. Available from: http://dx.doi.org/10.1016/S0006-3495(03)74449-X.

30. Bullerjahn JT, Sturm S, Kroy K. Theory of rapid force spectroscopy. Nat Commun. 2014;5:4463. Available from: http://dx.doi.org/10.1038/ncomms5463.

31. Dudko OK, Hummer G, Szabo A. Intrinsic rates and activation free energies from single-molecule pulling experiments. Phys Rev Lett. 2006 Mar;96(10):108101.

32. Getfert S, Reimann P. Optimal evaluation of single-molecule force spectroscopy experiments. Phys Rev E. 2007;76(5):052901. Available from: http://journals.aps.org/pre/abstract/10.1103/PhysRevE.76.052901.

33. Nguyen DH, Webb DJ, Catling AD, Song Q, Dhakephalkar A, Weber MJ, et al. Urokinase-type plasminogen activator stimulates the Ras/Extracellular signal-regulated kinase (ERK) signaling pathway and MCF-7 cell migration by a mechanism that requires focal adhesion kinase, Src, and Shc. Rapid dissociation of GRB2/Sps-Shc complex is associated with the transient phosphorylation of ERK in urokinase-treated cells. J Biol Chem. 2000 Jun;275(25):19382–19388. Available from: http://dx.doi.org/10.1074/jbc.M909575199.

34. Schlaepfer DD, Jones KC, Hunter T. Multiple Grb2-mediated integrin-stimulated signaling pathways to ERK2/mitogen-activated protein kinase: summation of both c-Src- and focal adhesion kinase-initiated tyrosine phosphorylation events. Mol Cell Biol. 1998 May;18(5):2571–2585.

35. Brami-Cherrier K, Gervasi N, Arsenieva D, Walkiewicz K, Boutterin MC, Ortega A, et al. FAK dimerization controls its kinase-dependent functions at focal adhesions. EMBO J. 2014 Feb;33(4):356–370. Available from: http://dx.doi.org/10.1002/embj.201386399.

36. Kholodenko BN, Demin OV, Moehren G, Hoek JB. Quantification of short term signaling by the epidermal growth factor receptor. J Biol Chem. 1999 Oct;274(42):30169–30181.

37. Hatakeyama M, Kimura S, Naka T, Kawasaki T, Yumoto N, Ichikawa M, et al. A computational model on the modulation of mitogen-activated protein kinase (MAPK) and Akt pathways in heregulin-induced ErbB signalling. Biochem J. 2003 Jul;373(Pt 2):451–463. Available from: http://dx.doi.org/10.1042/BJ20021824.

38. Hoops S, Sahle S, Gauges R, Lee C, Pahle J, Simus N, et al. COPASI–a COmplex PAthway SImulator. Bioinformatics. 2006 Dec;22(24):3067–3074. Available from: http://dx.doi.org/10.1093/bioinformatics/btl485.

39. Boerner RJ, Kassel DB, Barker SC, Ellis B, DeLacy P, Knight WB. Correlation of the phosphorylation states of pp60c-src with tyrosine kinase activity: the intramolecular pY530-SH2 complex retains significant activity if Y419 is phosphorylated. Biochemistry. 1996 Jul;35(29):9519–9525. Available from: http://dx.doi.org/10.1021/bi960248u.

40. Koo A, Nordsletten D, Umeton R, Yankama B, Ayyadurai S, García-Cardeña G, et al. In silico modeling of shear-stress-induced nitric oxide production in endothelial cells through systems biology. Biophys J. 2013 May;104(10):2295–2306. Available from: http://dx.doi.org/10.1016/j.bpj.2013.03.052.

41. Yee KL, Weaver VM, Hammer DA. Integrin-mediated signalling through the MAP-kinase pathway. IET Syst Biol. 2008 Jan;2(1):8–15. Available from: http://dx.doi.org/10.1049/iet-syb:20060058.
